# Fully automatic segmentation of glottis and vocal folds in endoscopic laryngeal high-speed videos using a deep Convolutional LSTM Network

**DOI:** 10.1371/journal.pone.0227791

**Published:** 2020-02-10

**Authors:** Mona Kirstin Fehling, Fabian Grosch, Maria Elke Schuster, Bernhard Schick, Jörg Lohscheller

**Affiliations:** 1 Department of Computer Science, Trier University of Applied Sciences, Schneidershof, Trier, Germany; 2 Department of Otorhinolaryngology and Head and Neck Surgery, University of Munich, Campus Grosshadern, München, Germany; 3 Department of Otorhinolaryngology, Saarland University Hospital, Homburg/Saar, Germany; Beijing University of Technology, CHINA

## Abstract

The objective investigation of the dynamic properties of vocal fold vibrations demands the recording and further quantitative analysis of laryngeal high-speed video (HSV). Quantification of the vocal fold vibration patterns requires as a first step the segmentation of the glottal area within each video frame from which the vibrating edges of the vocal folds are usually derived. Consequently, the outcome of any further vibration analysis depends on the quality of this initial segmentation process. In this work we propose for the first time a procedure to fully automatically segment not only the time-varying glottal area but also the vocal fold tissue directly from laryngeal high-speed video (HSV) using a deep Convolutional Neural Network (CNN) approach. Eighteen different Convolutional Neural Network (CNN) network configurations were trained and evaluated on totally 13,000 high-speed video (HSV) frames obtained from 56 healthy and 74 pathologic subjects. The segmentation quality of the best performing Convolutional Neural Network (CNN) model, which uses Long Short-Term Memory (LSTM) cells to take also the temporal context into account, was intensely investigated on 15 test video sequences comprising 100 consecutive images each. As performance measures the Dice Coefficient (DC) as well as the precisions of four anatomical landmark positions were used. Over all test data a mean Dice Coefficient (DC) of 0.85 was obtained for the glottis and 0.91 and 0.90 for the right and left vocal fold (VF) respectively. The grand average precision of the identified landmarks amounts 2.2 pixels and is in the same range as comparable manual expert segmentations which can be regarded as Gold Standard. The method proposed here requires no user interaction and overcomes the limitations of current semiautomatic or computational expensive approaches. Thus, it allows also for the analysis of long high-speed video (HSV)-sequences and holds the promise to facilitate the objective analysis of vocal fold vibrations in clinical routine. The here used dataset including the ground truth will be provided freely for all scientific groups to allow a quantitative benchmarking of segmentation approaches in future.

## Introduction

In current post-industrial societies a main part of the working population is reliant upon well-functioning communication skills. A prerequisite for efficient verbal communication is the production of a proper voice signal which constitutes the carrier signal of speech. Any impairment of the voice production process has a direct impact on the perceivability of speech affecting the communication ability. A cross-sectional survey study carried out by Roy et al. in 2005 showed a lifetime prevalence of a voice disorder of up to 29.9% interfering with verbal communication [1]. Work-related absences due to voice disorders as well as medical consultations causing significant socioeconomic costs. Therefore, the early diagnosis and effective therapy of voice disorders is of great importance.

The two opposing vocal folds within the larynx serve as voice generating structures. During voice production (phonation) they constitute a constriction for the exhaled respiratory airflow provided by the lung. Due to the interaction between the driving aerodynamic forces and myoelastic restoring forces of the tissue, oscillations of the vocal folds are provoked. Although the vocal fold vibration itself is a passive process, its vibration characteristics as e.g. the fundamental frequency *f*_0_ (pitch) and intensity can be altered by adapting the provided air pressure and laryngeal muscle activities [2]. Due to the different sizes of the laryngeal structures in males and females the fundamental frequency *f*_0_ of vocal fold vibrations is sensitive to gender. The mean *f*_0_ is around 120*Hz* for men and around 200*Hz* for women [3].

Understanding the underlying formation mechanism of voice disorders requires an in-depth investigation and analysis of vocal fold vibration patterns. In healthy subjects vocal fold vibrations are characterized by symmetric and highly periodic oscillations [2, 4, 5]. On the contrary, in the presence of voice disorders disturbances of the symmetric and periodic oscillation patterns arise induced by morphological asymmetries or inappropriate muscle tensions [6–8]. In order to quantify the degree of vibration disturbances the vocal fold (VF) oscillation patterns need to be investigated during phonation using laryngeal imaging techniques.

In clinical practice videostroboscopy is widely used for the examination of vocal fold (VF) vibrations [9]. Since the sampling rate of videostroboscopic systems is however far below the fundamental frequency of voice signals, they fail to adequately capture the real vocal fold (VF) vibration characteristics. Currently, laryngeal high-speed videoendoscopy (HSV) is the only technique to record the true intracyclic vibratory behavior of vocal folds [10–12]. Today’s HSV-systems operate at temporal resolutions of up to 20, 000 fps (frames per second) [13], facilitating a real-time analysis of the VFs vibrations. The high-speed recordings allow to derive various information about the spatio-temporal vibration characteristics and enable a profound analysis of periodic as well as highly disturbed, aperiodic vocal fold (VF) vibrations [10, 13–15]. HSV-systems therefore provide the basis for an objective quantification and diagnosis of voice pathologies (dysphonia) [16, 17] and likewise help to develop and improve biomechanical models of voice production [18–22].

### Analysis of vocal fold vibrations

In today’s clinical practice, diagnosis of voice disorders is commonly based on a subjective evaluation of stroboscopic video recordings [23]. Perceptually judging clinically relevant features as lateral amplitude and phase asymmetries, irregularities of oscillation cycles and the degree of vocal fold closure time is however a challenging and time consuming subjective task [24, 25]. Moreover, due to the subjectiveness of the rating the visual assessment is subject to inter- and intra-rater variability.

An objective clinical examination of laryngeal pathologies demands for a quantitative analysis of vocal fold dynamics. To achieve this, the time-varying opening (glottis) between the oscillating vocal folds is typically extracted from subsequent images of a high-speed video sequence, further denoted as glottal area waveform (GAW) [26]. From the *GAW*(*t*) time signal quantitative measures describing the stability of the vibration pattern in respect to its vibration amplitude and cycle-periodicity as well as information about the duration of vocal fold contact time can be derived [23, 27–30]. The GAW-analysis provides first valuable information about glottal vibration characteristics but does not enable lateral comparisons of the vibration pattern of the left and right vocal fold.

The individual vibration patterns of left and right vocal fold (VF) can be derived by extracting one-dimensional (1D) trajectories from the video data for each vocal fold individually [31]. The trajectory approach allows the detection of lateral vibration asymmetries but its application is restricted to a single vocal fold position [32, 33]. Further extended approaches achieve an analysis of the visible two-dimensional (2D) vibration patterns comprising information about the spatio-temporal dynamics along the entire vocal folds length [12, 34, 35]. Thus, on the basis of a 2D-analysis even information about the anterior-posterior vibration modes can be derived [12, 34, 36].

All above described approaches have in common that the initial analysis step requires a precise segmentation of the glottal area from the high-speed video data. Erroneous or imprecise segmentations result inevitably into invalid interpretations of the underlying vibration patterns and consequently into incorrect measures. Since high-speed videos comprise thousands of images, a manual segmentation of the image data is not feasible. Therefore, automated segmentation algorithms are needed allowing an accurate, robust and efficient segmentation of the glottal area.

### Image processing of laryngeal high-speed videos

Up to the present the quantitative analysis of vocal fold dynamics bases for the very most part on the segmentation of the glottal area from the digital high-speed videos. This is because the relatively dark glottal area is clearly silhouetted against the surrounding vocal fold tissue, which facilitates a proper glottal segmentation. In the recent years, various approaches for glottis segmentation have been proposed. Widely used approaches employ thresholding techniques [26, 37–41], which show however partially an insufficient performance, especially in case of low image quality [23]. Other approaches include techniques based on gray-level derivatives [42], seeded region-growing procedures [6, 43, 44], active contour models [45–47], or use watershed transform for the segmentation task [48].

A successful integration of a quantitative high-speed analysis system into clinical practice would preferably allow the identification of the best performing segmentation procedure based on a thorough literature review. For the following three reasons the systematic comparison of segmentation performances of different approaches is, however, problematic:

Firstly, approaches presented in literature are mainly developed on the basis of just a limited number of individual images (12 − 840 images per study) [44–46, 48] or high-speed video (HSV) sequences (1-90 HSVs per study) [26, 37, 42–46, 48]. Information about the real number of analyzed images is even missing in some studies [26, 37, 38, 42, 43]. Due to the limited or unspecified sample size it is difficult to assess if the used image data really constitute representative samples as expected in clinical practice. Thus, the generalization of statements concerning segmentation performances is limited.

Secondly, the performances of the segmentation approaches are frequently just subjectively assessed [26, 37, 38, 42–46, 48] and quantitative comparisons to a ground truth segmentation do hardly exist. So far, to best of our knowledge there are only two studies where the segmentation accuracies were quantitatively evaluated. Within the first study Schenk et al. used salient regions and 3D geodesic active contours for glottis segmentation [47]. The Dice Coefficient (DC) was applied to quantify the segmentation accuracy. For evaluation purposes 25 individual frames were randomly chosen from a variety of HSVs. Due to the limited number of evaluated images the generalization of the presented segmentation performance (median $\tilde{DC}\;=\;0.76$) is however quite limited. Within the second study Lohscheller et al. presented a semi-automatic region-growing approach which was validated on a large-scale clinical dataset comprising 372 high-speed video recordings [6]. From each video a sequence comprising 500 subsequent frames was processed resulting into 186, 000 segmented images. From these data 630 images were randomly selected for evaluation purposes. The precision of the segmentation results was quantitatively measured by comparing computed positions of four anatomical landmarks—namely the anterior and the posterior ending of the glottal area, as well as the medial glottal positions—to manual segmentations of ten experts, which served as gold standard. Totally, 25, 200 manually segmented landmark positions were evaluated. It was shown that the precision of the computed landmarks (1.2 to 3.5 pixels) was at least as good as the variability of the manual segmentations (3.2 to 3.4 pixels) of the ten experts.

Thirdly, up to the present there is no established freely-available scientific reference high-speed dataset, which can be used to compare different approaches with each other in an objective way. The lack of quantitative data aggravates a profound identification of a current gold standard concerning the computerized segmentation of high-speed videos.

Besides the segmentation of the glottal area likewise the segmentation of the vocal folds tissue itself would be of great clinical interest to identify automatically for instance vocal fold inflammation. Due to the wide variability in individual shape, size, color and reduced contrast the automatic segmentation of vocal folds tissue is however much more challenging than the segmentation of the glottal area. To the best of our knowledge, the segmentation of oscillating vocal fold tissue from high-speed videos has not been reported in literature yet.

### Neural networks

In the recent years, deep learning has been enhancing the performance of multiple computer vision applications like object detection [49, 50], classification [51–54], and segmentation problems [55, 56]. Advances became particularly possible thanks to the development of deep Convolutional Neural Networks (CNNs) [57], which were introduced in 1989 [58]. Popular Convolutional Neural Network (CNN) architectures used for image classification are the *AlexNet* [51], the *ConvNet* [59], the *GoogLeNet* [60], the *ResNet* [61], and the *SegNet* [62]. Also in the field of medical image analysis, advances have been made by applying artificial Neural Networks (NNs) [63–66], which learn features directly from medical images. Neural Networks were for example used for skin cancer classification [54], segmentation of retinal optical coherence tomography scans [56], and the classification of lung patterns for interstitial lung diseases [67].

In 2015, Ronneberger et al. proposed the *U-Net* architecture [68], which is nowadays a widely used network architecture from the encoder-decoder class for segmentation purposes. By combining high resolution features from the contracting path (encoder) with upsampled features from the expanding path (decoder), it allows the segmentation of even detailed image structures on a fine scale. The *U-Net* has been proven to be appropriate for various semantic segmentation tasks. Ronneberger et al. initially used it for segmentation of neuronal structures in electron microscopy stacks as well as for segmentation of HeLa cancer cells in differential interference contrast microscopy images [68]. Amongst others, it was further used for retinal vessel segmentation [69], for pancreas segmentation in computer tomography images [70], and segmentation of radical prostatectomies from histological images [71].

Although the application of deep CNNs has achieved highly reliable results for various semantic segmentation tasks on medical images, CNNs have hardly been investigated on their suitability for the analysis of laryngeal high-speed videos. Up to the present only very few publications dealing with the segmentation of laryngeal structures using artificial Neural Networks can be found in literature [72–74]. The few presented approaches have in common that the images of video sequences are processed independently from each other. Thus, they apply Convolutional Neural Network (CNN) architectures as U-Net, SegNet, ENet, or ErfNet for segmentation of 2D images and concatenate the individual segmentation results subsequently. Here, stroboscopy and video recordings with a frame rate of 25 frames per seconds are used as data material [75]. Up to the present there is no work dealing with the segmentation of the fast oscillating vocal folds tissue from high-speed video recordings using CNNs. Likewise, to the best of our knowledge no approaches have been presented which take advantage of the spatio-temporal context of a high-speed video.

Suitable network architectures for the propagation of temporal information through a network are Recurrent Neural Networks (RNNs), which contain feed-back connections to propagate information from one cell to another making it particularly suitable for processing time signals such as video recordings [75]. An improvement over Recurrent Neural Networks (RNNs) can be achieved by Long Short-Term Memory Networks (LSTMs), which are capable of learning even long-term dependencies [76]. A variety of publications report the successful application of LSTMs for segmentation tasks, in which the spatio-temporal information is propagated through the network to enhance the segmentation quality [77–80]. Besides LSTM there are Gated Recurrent Units (GRU), which are likewise used to process time varying data [81].

In this work, we present for the first time a fully automatic glottis and vocal fold tissue segmentation procedure based on an extended version of the *U-Net* architecture, which provides single image segmentation. As high-speed videos represent a time signal, we integrate LSTM and GRU cells respectively at different positions within the contracting and expanding path of *U-Net* architecture to propagate temporal information throughout the network. We investigate and compare extensively the performance of eighteen different network configurations in combination with different data preprocessing steps in order to identify the best performing network architecture. Further, we investigate whether segmentation accuracy can be enhanced by data augmentation. The segmentation accuracy of the best performing network is evaluated in detail. It will be shown that once the Convolutional Long Short-Term Memory Network (CLSTM) is trained, it holds the promise to overcome the limitations of current glottis segmentation approaches which require manual user intervention. Furthermore, it will be demonstrated for the first time that even oscillating vocal fold tissue can be automatically segmented from high-speed videos. The here used dataset, including the ground truth and segmentation results, will be provided freely for all scientific groups to allow quantitative comparison of different types of segmentation approaches. Further information can be found at www.hochschule-trier.de/go/quantitative-laryngoscopy.

## Materials and methods

### Clinical data

**Fig 1:**
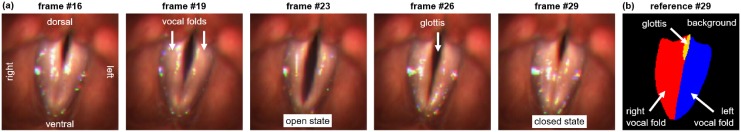
Laryngeal high-speed video data. (a) Frames from a single oscillation cycle of a healthy female subject (26 yrs, *F*_0_ = 215 Hz). (b) Reference segmentation for the last frame (# 29).

For the development and training of the system, we use clinical data obtained from the *Department of Otorhinolaryngology and Head and Neck Surgery* at the *University of Munich (Munich, Germany)* and the *Department of Otorhinolaryngology* at the *Saarland University Hospital (Homburg/Saar, Germany)*. Ethical approval was obtained from the local ethics committees (*Ethikkommission bei der Medizinischen Fakultät der LMU München* and *Ethik-Kommission bei der Ärztekammer des Saarlandes*) and the participants gave written consent prior to participation. All video recordings were captured using the rigid endoscopy system *HRES ENDOCAM 5562* from *Richard Wolf GmbH (Knittlingen, Germany)* that captures HSVs at a spatial resolution of 256 × 256 px with a frame rate of 4, 000 fps. To evaluate whether the presence of pathologies, i.e. a polyp, influences the segmentation accuracy, videos from 130 subjects were used comprising *N*_*H*_ = 56 recordings from healthy (#m: 21, #f: 35) and *N*_*P*_ = 74 recordings from pathologic subjects (#m: 39, #f: 35). The pathologic subjects were further subdivided into two groups. The group *organic* (*N*_*O*_ = 37) comprises recordings from 22 polyps (#m: 12, #f: 10) and 15 carcinomas (#m: 10, #f: 5). Dysphonia without neoplasm were summarized under the term *functional* (*N*_*F*_ = 37). This group contains 17 subjects diagnosed with muscle tension dysphonia (#m: 9, #f: 8) and 20 subjects diagnosed with paresis (#m: 8, #f: 12). All subjects were examined during sustained phonation of the vowel /ae/ at comfortable pitch and loudness for at least 1 s. From each of these HSVs, a sequence of 100 frames was extracted for further analysis. Fig 1(a) exemplarily shows five frames extracted from a single oscillation cycle of a healthy subject. The vocal folds as well as the intermediate time-varying opening (glottis) are subject of the segmentation approach presented here.

### Ground truth segmentations

The supervised training and the evaluation of the different CNN architectures require corresponding reference segmentations serving as Ground Truth (GT). For this purpose the following four classes were defined: *glottis*, *left vocal fold*, *right vocal fold*, and *background*. For all 13, 000 images comprised within this study, reference segmentations were obtained by the following two-stage procedure: At first, the glottal area was segmented using the supervised region-growing procedure presented by Lohscheller et al. [6]. The approach was chosen since it is the only reported procedure whose segmentation precision has been quantitatively investigated within an extensive study [6]. Furthermore, it allows the computation of four relevant landmark positions (anterior and the posterior ending of the glottal area, left/right medial glottal positions) which are later used for evaluation purposes. All glottal segmentation results were visually inspected and manually corrected if required. In the second step, the ground truth segmentations of the vocal fold tissue was generated. Since up to the present there are no computerized procedures, the vocal fold tissue segmentation was performed completely manually. All other remaining pixels were considered as *background*. As a result of this two-stage procedure each pixel was assigned to one of the four classes constituting the Ground Truth (GT). Fig 1(b) exemplarily shows a reference segmentation for the last frame of the presented sequence.

### Datasets

In total, 130 HSVs comprising 13,000 individual images were used for training, validating and testing. Using a stratified sampling, 100 recordings were randomly selected serving as training data, while 15 videos were selected to build the validation and test dataset respectively. Each dataset contains recordings from healthy subjects as well as pathologic subjects with functional impairments and organic lesions. The training data include 50 healthy and 50 pathologic subjects, while the functional and organic subgroups were balanced. Validation and test datasets include each 15 recordings in total with 3 recordings for each group. The quantification of the segmentation accuracy is thus performed on the basis of 1,500 test images.

### Network architectures

**Fig 2:**
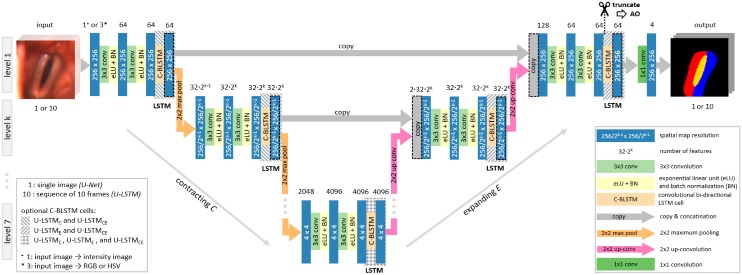
Schematic diagram of the applied Convolutional Neural Network architectures. Individual grayscale, RGB or HSV frames of the high-speed video sequence to be analyzed serve as input. Feature-maps’ spatial dimensions are provided inside the boxes, while the number of extracted feature maps is shown above. During the contracting path the pixel information is downsampled. Extracted features are further propagated to higher levels in the expansive path and concatenated, which is represented by arrows. B-CLSTM cells propagate the temporal information through the network. A 256 × 256 px sized segmentation result is returned, where the value ‘1’ represents the class *glottis*, while the *right* and the *left vocal fold* are indicated by ‘2’ and ‘3’ respectively. The best performing network operates on sequences of ten native RGB video frames as input and has a depth of five levels with C-BLSTM cells in contracting and expanding path.

For the here aspired glottal area and VF tissue segmentation the U-Net, proposed by Ronneberger et al., serves as basis architecture [68]. As shown in Fig 2 the U-Net represents an encoder-decoder approach and basically consists of a contracting path for downsampling and an expanding path for upsampling. By concatenating the features from both paths, even fine-scale segmentation maps can be achieved. In our work we refine the original architecture by using exponential linear units (eLU) instead of rectified linear units (ReLU) for activation as suggested by Clevert et al. [82]. Furthermore, batch normalization is introduced as suggested by Ioffe and Szegedy [83] as well as padded-convolutions to allow for identical input and output size of the images. In this work, the refined architecture will further be referred to as U-Net. The U-Net can be parameterized by the number of downsampling and upsampling levels which directly influences the segmentation performance. Depending on the number of applied levels *L* the particular architecture is denoted as U-Net_*L*_.

Vocal fold vibrations as well as the glottal opening and closing process constitute continuous motion processes. Due to the high sampling rate of the camera system, there is little change of the shape and location of these structures in-between subsequent images. Therefore, knowledge about their positioning’s obtained from an image at a certain time constitutes valuable information which can be further used for a proper segmentation of temporally adjacent images. In this work the incorporation of temporal information is achieved by integrating additional convolutional Long Short-Term Memory Network (LSTM) cells [76, 84] into the U-Net architecture. Since the segmentation is performed on video sequences loaded from hard-disk the application of even bi-directional LSTM cells (C-BLSTM) is feasible, which process information in both temporal directions [78]. In this work C-BLSTM layers are implemented by stacking two C-LSTM layers (3 × 3 convolutions) on-top of each other. The first layer processes the video sequence in forward the other in backward direction. The concatenation of the feature maps of both layers constitute the output of the resulting C-BLSTM layer.

According to Gao et al. [79] the C-BLSTM layers can be integrated at different positions within the U-Net architecture. To identify the best performing integration, C-BLSTM layers are optionally positioned in the contracting (*C*), expanding (*E*) or in both paths (*CE*) simultaneously (see Fig 2). Depending on the positioning of the LSTM-layers the resulting architectures are denoted as $\text{U}-{\text{LSTM}}_{L}^{C,E,CE}$. In the above described architectures the weights of the U-Net and the LSTM-layers are trained simultaneously which makes the training process computationally more demanding.

Besides integrating C-BLSTM layers directly within a U-Net they can also be added as an individual LSTM-network on top of an already trained U-Net. Thus, both networks can be trained independently from each other which reduces the training workload. In our work the input of the LSTM-network consists of the 64 feature maps of the first level. For this purpose the U-Net gets truncated at the particular position as indicated in Fig 2. The architecture with an LSTM add-on (*AO*) strategy is further referred to as $\text{U}-{\text{LSTM}}_{L}^{AO}$.

Frequently GRU cells are used to process time-varying data. To investigate whether GRU cells can further enhance segmentation accuracy over C-BLSTM layer, the best performing U-LSTM architecture is compared to an equivalent configuration equipped with GRU cells and denoted as U-GRU.

### Identifying the best performing network architecture

In order to identify the most suitable network for high-speed video segmentation, the performances of the different architectures were compared to each other. Particular focus was on the performance of the U-LSTM networks which integrate temporal information into the segmentation process. Since the correct segmentation of the glottis is the primary goal for later clinical voice analysis, the Sørensen–Dice coefficient (DC) computed for the class *glottis* served as metric to assess the segmentation performances. Besides the network architecture likewise the image representation (color space) as well as preprocessing of the input data influence the segmentation performance. In order to determine the optimal combination of network architecture, color space representation and data preprocessing the following hierarchical selection strategy was applied:

*(1)* Within the U-Net_*L*_, that constitutes the basis for all investigated architectures, the number of levels *L* represents a free design parameter which has a direct impact on the segmentation performance. The number of U-Net levels can be freely defined within a certain interval that depends on the spatial resolution of the image data. Here, the performances of five different architectures with *L* = [3, 4,.., 7] levels were compared to each other. The RGB color space was used to represent the image input data. The DC—computed for the class *glottis* of the test dataset—served as metric to assess the segmentation performance. Selection of the best performing U-Net_L*_ architecture (indicated by the asterisk) was made on the basis of the highest mean Dice Coefficient. ANOVA (*α* = 0.05) in combination with bonferroni corrected pairwise post-hoc t-tests were performed for identification of significant differences between the segmentation performances of the different networks.

*(2)* Based on the best performing U-Net_L*_ architecture the influence of the following three image representations (IR) on the segmentation performance was investigated: original RGB color space (*IR* = *RGB*), HSV color space (*IR* = *HSV*), and grayscale intensity image (*IR* = *I*). Again, the selection of the best performing configuration, denoted as $\text{U}-\text{Net}{}_{L^{*}}^{IR^{*}}$, was made in respect to the mean value of the Dice Coefficient. Likewise, ANOVA (*α* = 0.05) with pairwise post-hoc t-tests (bonferroni correction) were applied for statistical analysis.

*(3)* Based on $\text{U}-\text{Net}{}_{L^{*}}^{IR^{*}}$ the impact of normalization (*Norm*) of the input image data was investigated. For this purpose, the z-transform was applied before training to standardize the image data of each training batch (*Norm* = *zbatch*). The results were compared to the segmentation performance obtained without pre-processing (*Norm* = *none*). The best performing configuration (highest mean value of the Dice Coefficient) is denoted as $\text{U}-\text{Net}{}_{L^{*}}^{IR^{*},Norm^{*}}$. Statistical analysis was made using a t-test (*α* = 0.05).

*(4)* Finally, the selected best performing U-Net configuration was used to incorporate temporal information by integrating C-BLSTM cells at different positions (*Pos*). Here, four different strategies were investigated: Placing the C-BLSTM layers in the contracting (*Pos* = *C*), the expanding (*Pos* = *E*), in both paths simultaneously (*Pos* = *CE*), or adding the C-BLSTM layers on top of the pre-trained U-Net (*Pos* = *AO*). The configuration with the highest mean DC value was identified and is further denoted as U – LSTM^Pos*^. A final ANOVA (*α* = 0.05) with pairwise post-hoc t-tests (bonferroni correction) was applied for statistical analysis.

Additionally, the identified U-LSTM^Pos*^ configuration was compared to an equivalent configuration equipped with GRU cells, which is denoted as U-GRU^Pos*^. Further, data augmentation was applied on the overall best performing Convolutional Neural Network (CNN) configuration. For this purpose the training dataset was doubled to 20,000 images by randomly rotating the individual sequences and corresponding masks at rotation angles ∈ [−20°, 20°]\0°. That followed cropping and rescaling was applied, such that no padding was need and the resulting frames preserve the initial size of 256 px × 256 px.

### Training

In total eighteen CNNs were trained on the input images of the training dataset and corresponding Ground Truth (GT) segmentations. The U-Net architectures without C-BLSTM cells were trained with a batch-size of 10 images and 20 epochs having 1,000 iterations each. As loss function cross-entropy with pixel-wise softmax-layer was used. The loss is reduced by the Adam optimizer [85] with an initial learning rate of 10^−4^, and the hyper-parameters *β*_1_ = 0.9, *β*_2_ = 0.999, and *ϵ*: 10^−8^. A Xavier Initialization of the Convolutional Neural Network (CNN) weights was done using Gaussian distribution with a standard deviation of $\sqrt{2/N_{avg}}$ to guarantee that all layers have roughly same variance, with *N*_*avg*_ indicating the average sum of input *N*_*in*_ and output connections *N*_*out*_ [86]. Overfitting was avoided by the application of dropout layers with *probability*: 0.6.

The Convolutional Neural Network (CNN) architectures containing C-BLSTM or GRU cells were trained on sequences of 10 consecutive frames with a batch size of 1 video sequence for 40 epochs.

### Testing

To receive the segmentation result for evaluating the segmentation performance, the final feature maps of the different architectures were assigned to the four classes by applying subsequently four 1 × 1 convolutions in combination with a pixel-wise softmax layer to calculate the class probabilities. The output channel with maximum probability defines the corresponding class.

For evaluation purposes the trained U-Net architectures that process images independently from each other, this means without integration of temporal information, were applied to all images of the test dataset. In contrast, for the LSTM and GRU architectures batches of 10 subsequent frames were presented to the Neural Network producing 10 output segmentations each, which were further considered to assess the segmentation performance. This approach facilitates smooth and continuous segmentation results over time and can only be applied networks comprising temporal information.

Training as well as testing of the Convolutional Neural Network (CNN) was done on the High Performance Computer ‘Elwetritsch’ (University of Kaiserslautern, Germany) either on a Host with *Intel XEON SP 6126* and up to two 16 GB *NVIDIA Tesla V100* GPUs or on the *NVIDIA DGX-2* system. *NVIDIA CUDA version 10.0-v7*, *TensorFlow version 1.13.1* [87] and *Python 3.6.8* was used.

### Quantifying the segmentation quality of the best performing network

The following analysis is conducted on the best performing network configuration, to quantify in detail the overall quality and precision of the obtained segmentation results. In the context of quantitative quality assessment of high-speed video segmentations the following two different aspects are of particular interest.

#### (1) Accuracy of glottal area segmentation over time

**Fig 3:**
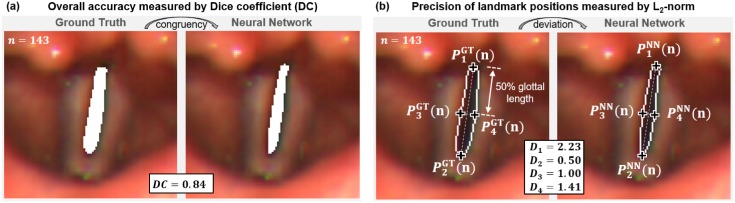
Quantifying the segmentation accuracy by comparing ground truth and Neural Network segmentations. (a) Overall segmentation accuracy of the glottis was measured using the Dice Coefficient. (b) Spatial segmentation precision was quantified by computing the deviation *D*_*i*_(*n*) at the four landmark positions *P*_*i*_(*n*) using the *L*_2_-Norm.

Firstly, the overall time course of the glottal area needs to be segmented correctly since faulty segmentations of individual images would make a further analysis of the glottal time signal impossible. To measure the overall congruency between the segmented glottal area and the ground truth the Dice Coefficient *DC*(*n*) [88] was computed individually for all frames *n* of the videos contained in the test dataset according to

$$\begin{array}{c} DC\left(n\right)=\frac{2|GT(n)\cap NN(n)|+\epsilon }{|GT(n)|+|NN(n)|+\epsilon }, \end{array}$$ (1)

where *GT*(*n*) denotes the Ground Truth and *NN*(*n*) the Neural Network segmentation. The value *ϵ* = 2.2204 ⋅ 10^−16^ is added to avoid division by zero in case that both segmentations *GT*(*n*) and *NN*(*n*) contain no *glottis* pixels which may happen during complete glottal closure. The Dice Coefficient is in the range *DC*(*n*) ∈ [0, 1] with higher DC values indicating superior segmentation congruency. An exemplary video frame from the test dataset with corresponding GT and NN segmentation and calculated Dice Coefficient (DC) is shown in Fig 3(a). Segmentation congruency of the considered sequence was investigated using the mean as well as the standard deviation of *DC*(*n*) computed for all images. According to that, likewise the Dice Coefficients for the left and right vocal folds were computed to asses the respective segmentation quality.

#### (2) Precision of anatomical landmark positions

The analysis of the time varying deflections of vocal folds demands a precise segmentation of the vocal fold edges. Since the Dice Coefficient represents just a global measure it does not allow conclusions about the segmentation precision at specific positions. In order to quantify the segmentation accuracy at relevant vocal fold positions, the spatial deviation between Ground Truth (GT) and NN segmentation was computed at four anatomical landmarks *P*_*i*_(*n*) with *i* ∈ [1, 4] located at the VFs edges according to the study of Lohscheller et al. [6]. The considered landmarks are defined in Fig 3(b), which are namely the dorsal (*P*_1_(*n*)) and the ventral glottal ending (*P*_2_(*n*)), as well as the right (*P*_3_(*n*)) and left medial glottis positions (*P*_4_(*n*)). The location of the medial line is defined on the Ground Truth (GT) segmentation and transferred to the NN segmentation to allow direct comparisons. The spatial deviation *D*_*i*_(*n*) between both segmentations is measures by the *L*_2_−*Norm* as

$$\begin{array}{c} D_{i}\left(n\right)={∥P_{i}^{GT}\left(n\right)-P_{i}^{NN}\left(n\right)∥}_{2}. \end{array}$$ (2)

## Results

Eighteen different network configurations were investigated to identify the best performing architecture. As metric the Dice Coefficient (DC) computed for the glottal area was used since the proper segmentation of the glottis is the most relevant outcome for following voice analysis. Subsequently, segmentation accuracy of the best performing network is investigated in detail.

### Identification of the best performing network architecture

**Fig 4:**
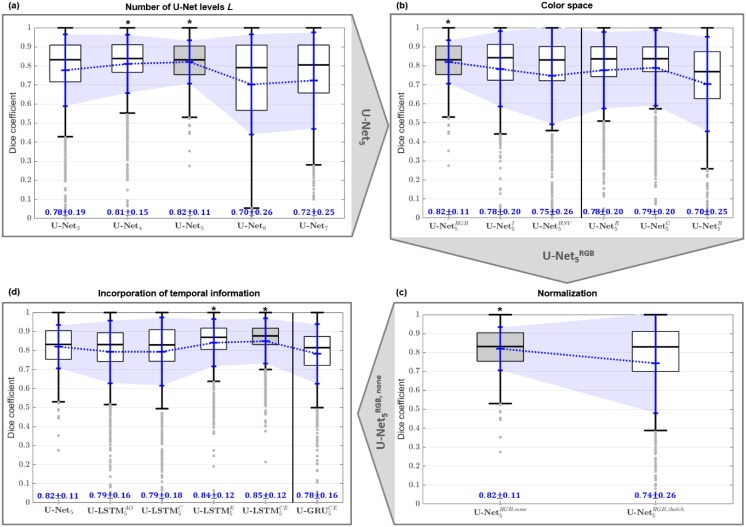
Identification of the best performing network architecture based on a hierarchical selection strategy. Impact of (a) number of U-Net levels, (b) color space representation, (c) normalization, and (d) positioning of C-BLSTM layers and GRU cells. Results show the distribution of the Dice Coefficients obtained from 1,500 test images. Mean DC indicated by blue ‘+’. The best performing configuration is highlighted in gray.

The best performing network configurations was identified on the basis of an ordered selection strategy. For this purpose, the impact of the number of U-Net levels, color space, normalization and different positions of C-BLSTM layers were consecutively evaluated. For each configuration the epoch was identified yielding the best results in respect to the validation data. The selection of the best performing network was subsequently done on the performance of the test dataset using the network weights of the respective epoch. The so selected configurations were used for the next evaluation step. Differences between the configurations in respect to the test dataset were investigated using statistical analysis (ANOVA, t-test). In the following the results of the stepwise selection strategy are presented which are further summarized in Fig 4.

(a) The five level U-Net_*L* = 5_ network showed the best performance in respect to the number of levels with a mean Dice Coefficient of $\bar{DC}=0.82\pm 0.114$ (± indicates the standard deviation). Statistical analysis revealed significant differences to the *L* = 3, 6, 7 level architectures. The four level network showed a comparable performance of $\bar{DC}=0.811\pm 0.15$ but an increased standard deviation of approximately 33%. For a higher number of levels the segmentation quality dropped clearly. Therefore, the U-Net_5_ network was identified as best performing architecture and further used for the subsequent evaluation step.

(b) In respect to different color spaces, the segmentations based on native RGB data showed the best performance ($\bar{DC}=0.82\pm 0.114$). ANOVA with post hoc t-tests revealed significant differences to the segmentation performances when using grayscale intensity images ($\bar{DC}=0.78\pm 0.20$) or the HSV color space ($\bar{DC}=0.75\pm 0.25$). To investigate whether one channel of the RGB color space is of outstanding importance for this particular segmentation task, the NN was additionally trained on intensity values of the individual color channels. While the R- and G-channel showed segmentation performance comparable to intensity images ($\bar{DC}=0.78\pm 0.20$ and $\bar{DC}=0.79\pm 0.20$ respectively), performance for the B-channel was reduced ($\bar{DC}=0.70\pm 0.25$). The use of native RGB data likewise delivers the most robust segmentation results reflected by the lowest value of the standard deviation of the Dice Coefficient.

(c) As best performing configuration the ${\text{U-Net}}_{5}^{RGB}$ was used to investigate potential improvements by applying z-normalization as data preprocessing step. Statistical analysis (t-test) revealed, that normalization has no positive impact on the segmentation performance. In our experiments with a mean of $\bar{DC}=0.74\pm 0.26$ batch standardization even worsen the segmentation accuracy. Thus, the segmentation performance of the ${\text{U-Net}}_{5}^{RGB,none}$ served as reference for the next evaluation step.

(d) Finally, the impact of incorporating temporal information into the network was investigated. For this purpose C-BLSTM layers were added at four different positions to the ${\text{U-Net}}_{5}^{RGB,none}$ network. Statistical analysis revealed, that placing C-BLSTM layers either into the expanding path ($\bar{DC}=0.84\pm 0.123$) or simultaneously into the contracting and expanding paths $\bar{DC}=0.85\pm 0.119$ lead to the best segmentation results.

As GRU cells are likewise used to process time varying data, additionally a U-Net configuration equipped with GRU cells in contracting as well as expanding path was evaluated. However, this $\text{U}-{\text{GRU}}_{5}^{CE}$ did not outperform segmentation accuracy achieved by the best U-LSTM ($\bar{DC}=0.78\pm 0.16$). Further, data augmentation was applied to the best performing configuration. However, training the ${\text{U-LSTM}}_{5}^{CE}$ on the expanded dataset comprising 20,000 images in total has not enhanced segmentation performance ($\bar{DC}=0.83\pm 0.14$).

**Fig 5:**
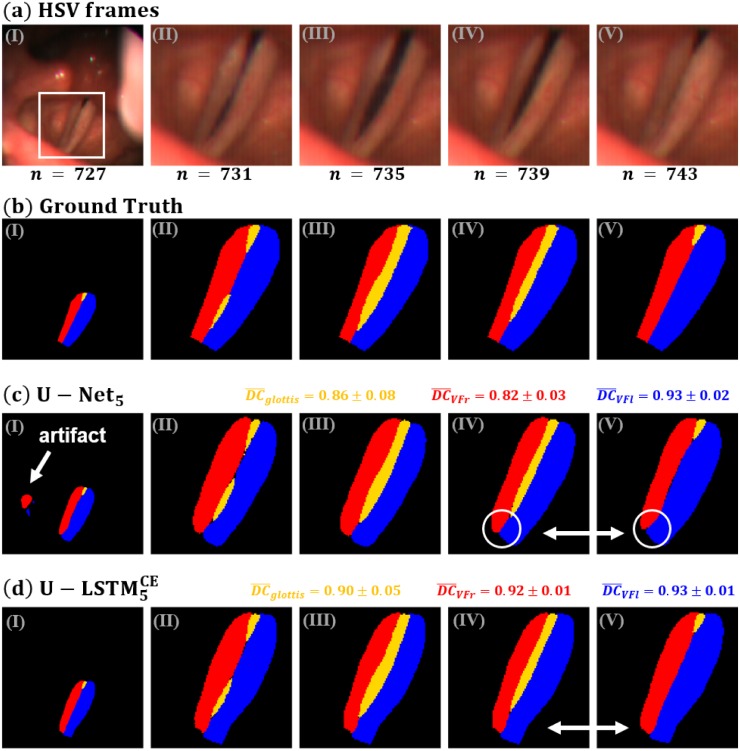
Comparison of segmentation results of individual images within a single subject. (a) Images of test dataset, (b) Ground Truth, (c) U-Net segmentation, (d) U-LSTM segmentation. The Dice Coefficients given for each class represent the mean and standard deviation of the entire sequence.

In order to get better understanding of the benefit of the applied LSTM-layers, Fig 5 exemplarily compares the segmentation results of the best performing U-Net (c) with the best performing U-LSTM (d). Likewise the original image data (a) and the ground truth (b) are presented. For both networks the mean Dice Coefficients including the standard deviations are given, which were computed for each class independently.

Within the first segmented frame of the U-Net, segmentation artefacts are visible marked by the white arrow. Contrarily, in the U-LSTM segmentation results the artifacts do not occur. Here, due to the integration of temporal information into the architecture the network was trained in such a sense, that intermittently occurring artifacts appearing far away from the glottis cannot belong to the classes *left* and *right vocal fold* which suppresses such artifacts.

In the segmentations of the U-Net it can be further seen, that the anterior parts of the vocal folds (white circles) deform quite heavily during the sequence. This results because the images are processed independently by the U-Net. Contrarily, the deformations of the vocal fold tissue segmented by the U-LSTM are much smoother over time. Here, the network learned that vocal folds do not change their outer shape that rapidly during short time intervals. The better temporal consistency of the segmentation results obtained by the U-LSTM is likewise reflected by the high values of the Dice Coefficient, which outperform the U-Net architecture.

Since the ${\text{U-LSTM}}_{5}^{CE}$ shows the highest mean Dice Coefficient, and due to its superior performance in respect to producing temporal consistent segmentations, the architecture was selected as best performing network configuration.

### Segmentation quality of the best performing network

In the following the results of an in-depth analysis of the segmentation performance achieved by the ${\text{U-LSTM}}_{5}^{CE}$ architecture is presented. In this context, the segmentation quality concerning the overall time course of the glottal area and the precision of landmark segmentations will be shown in detail.

#### (1) Accuracy of glottal area segmentation over time

**Fig 6:**
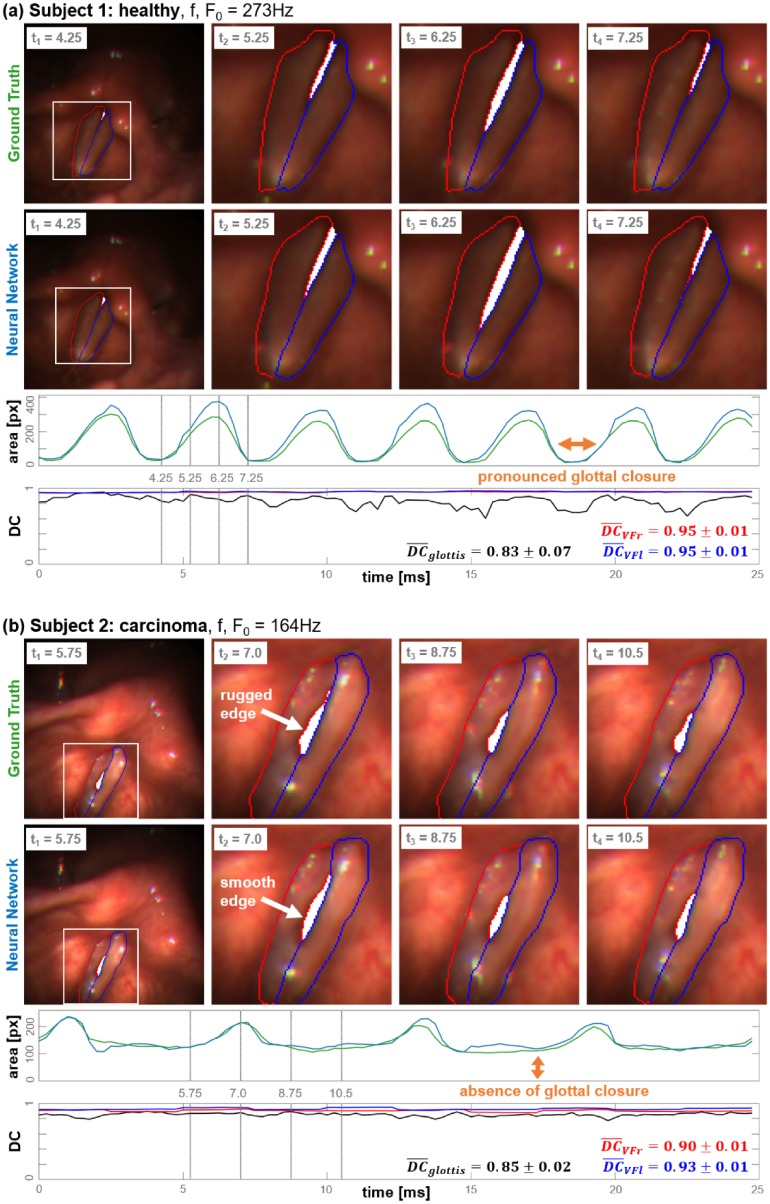
Accuracy of glottal area segmentation over time. (a) Healthy subject. (b) Pathologic subject (carcinoma). From top down: HSV frames extracted from a single oscillation cycle. Upper row: GT segmentations, Lower row: NN segmentations. The upper graph shows the glottal area waveforms (green: GT, blue: NN) while the lower one represents the Dice Coefficients which are computed for each class individually (red/blue: *right/left vocal fold*, black: *glottis*).

The ${\text{U-LSTM}}_{5}^{CE}$ was applied to segment all sequences of the test dataset comprising 100 frames each. For one healthy and one pathologic subject (carcinoma) the obtained results are exemplarily presented in Fig 6. For both cases four images were exemplarily selected out of a single oscillation cycle in which the segmentation results are shown as overlay. Furthermore, for each image of the sequence the size of the glottal area was computed (number of pixels of the class *glottis*) which is displayed as blue curve in the graph below. In literature this curve is referred to as glottal area waveform (GAW). To assess the quality of the results likewise the Ground Truth (GT) is displayed serving as reference. Finally, for each class the Dice Coefficients between NN and GT segmentations are computed which are plotted for the entire sequence in the lower graph.

In both cases the time course of the GAW obtained by the NN agrees very well with the Ground Truth. No serious erroneous segmentations occurred within these sequences. For the healthy subject the NN tends to slightly overestimate the total size of the glottal area even though these differences are barely visible in the individual frames (Fig 6). For the carcinoma case, the GAW signal is determined with a remarkably high precision. Just very slight differences to the GT segmentation can be perceived. For instance the edge of the affected right vocal fold (VF) is slightly smoother compared to Ground Truth (GT) segmentation.

For the pathologic case the Dice Coefficient (DC) for the glottal area is almost constant throughout the entire sequence $\bar{DC}\;=\;0.85\;\pm \;0.02$. For the healthy subject the mean $\bar{DC}$ is slightly reduced while the standard deviation is more than three times increased ($\bar{DC}\;=\;0.83\;\pm \;0.07$). The relatively pronounced changes of the Dice Coefficient over time are caused in the healthy subject by the almost complete glottal closures during the closing phases, which do not occur in the pathological case. If the glottal area of the Ground Truth comprises just a few pixels even marginal deviations of the NN segmentation lead to significantly reduced Dice Coefficients.

**Fig 7:**
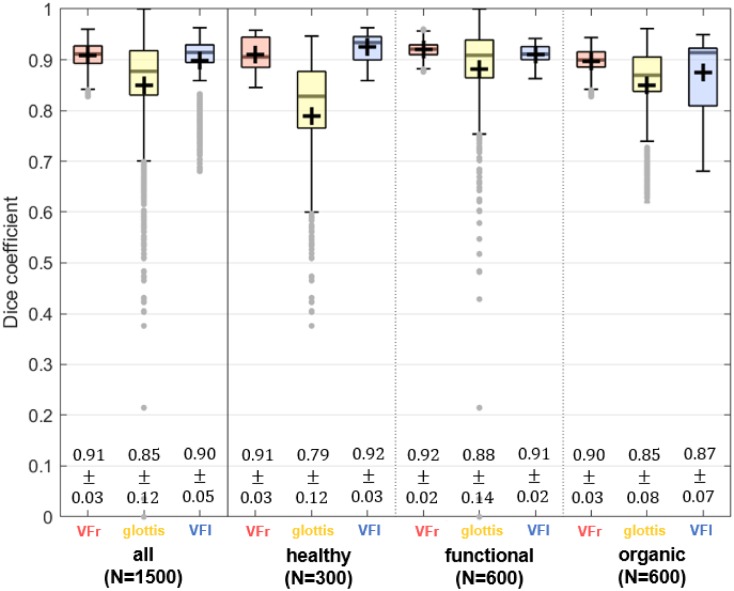
Segmentation accuracy measured by the dice coefficient for the test dataset. Dice Coefficients are individually displayed as boxplots for the classes *glottis*, *left VF*, and *right VF*. Left: Entire dataset. Right: Clinical groups *healthy*, *functional*, and *organic*. The mean values and standard deviations are given below.

The Dice Coefficients computed for the three classes for the entire test dataset are presented as boxplots in Fig 7 (left). For all 1,5000 images the average DC amounts for the *glottis* class $\bar{DC}=0.85\pm 0.12$. For the classes *right VF* ($\bar{DC}=0.90\pm 0.05$) and *left VF* ($\bar{DC}=0.91\pm 0.03$) the segmentation accuracy was even higher. To investigate whether the presence of pathologies influence the segmentation accuracy, the Dice Coefficients are subdivided into the clinical groups *healthy*, *functional*, and *organic* (Fig 7, right). The mean DCs of all groups exceed the value of 0.8 except for *glottis* class of the healthy subjects. As shown in Fig 6 healthy voices are characterized by a distinct glottal closure. Therefore, in a variety of images of the healthy group the glottal area becomes small. For the healthy group the mean size of the glottal area amounts only $\bar{GAW}=\left(218.0\;\pm \;144.5\right)\;px$ while it is increased for the *functional* and for the *organic* groups. For these groups the glottal area amounts $\bar{GAW}=\left(454.4\;\pm \;388.4\right)\;px$ and $\bar{GAW}=\left(423.64\;\pm \;311.0\right)\;px$ respectively. Since the Dice Coefficient is systematically influenced by the size of the glottis, a reduced Dice Coefficient (DC) and does not necessarily indicate a lower segmentation accuracy.

#### (2) Precision of anatomical landmark positions

**Fig 8:**
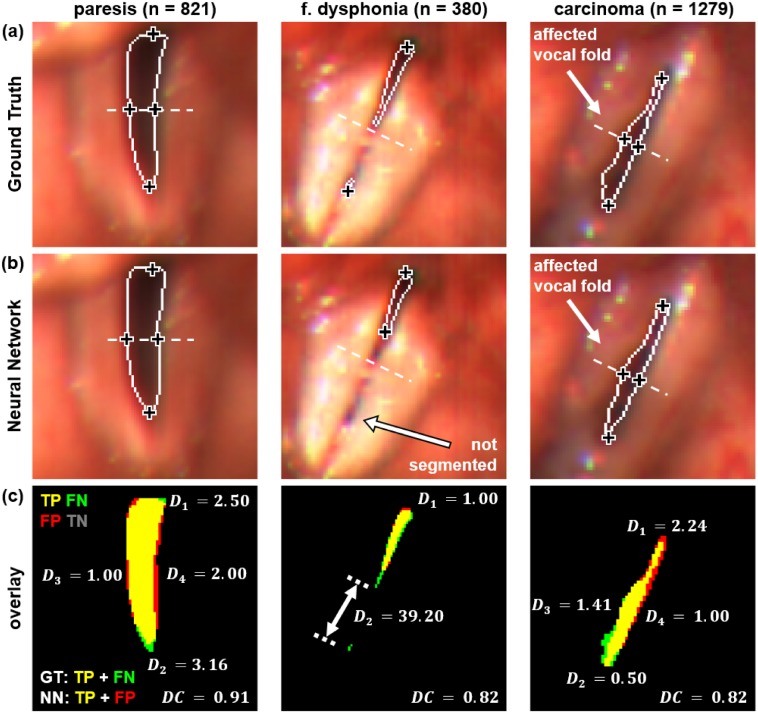
Comparison of landmark positions in three subjects (paresis, dysphonia, carcinoma). The landmarks *P*_1_-*P*_4_ are marked with ‘+’. (a) Ground Truth (GT) segmentation. (b) Best performing Neural Network (NN) segmentation. Within the functional dysphonia case the landmarks *P*_3_ and *P*_4_ could not be identified due to an insufficient segmentation. (c) Color-coded segmentation overlay. Yellow: True Positive (TP), green: False Negative (FN), red: False Positive (FP), black: True Negative (TN). Measured deviations *D*_1_-*D*_4_ are given in pixels. The high deviation for *P*_2_ = 39.2 px results from the not properly segmented ventral part of the glottis.

To investigate the spatial segmentation accuracy of the best performing network, its precision was measured at the four landmarks *P*_1_-*P*_4_ defined in Fig 3. To get a first impression of the accuracy of landmark detection, segmentation results obtained from three different clinical cases (paresis, dysphonia, carcinoma) are exemplarily presented in Fig 8. The positions of the landmarks *P*_1_-*P*_4_ are marked by ‘+’, while *D*_*i*_ indicates the deviations between Ground Truth (GT) and Neural Network (NN). To emphasize the differences between GT and NN segmentations more clearly a color-coded segmentation overlay is presented in the bottom row. The meaning of the color coding scheme is given in the caption. For all cases the spatial deviations of the landmarks *P*_1_, *P*_3_ and *P*_4_ between the Ground Truth (GT) and NN segmentations are below 3 pixels. Due to a closed state at the middle of the vocal folds, the medial landmarks *P*_3_ and *P*_4_ are not defined for the functional dysphonia case. The unsegmented glottal part is further responsible for the relatively high deviation of *D*_2_ = 39.20 px of the ventral landmark.

**Fig 9:**
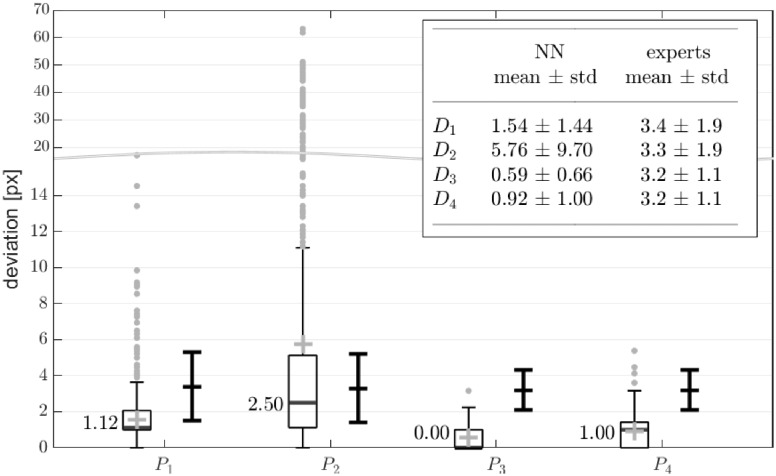
Accuracy of segmented landmarks P_1_-P_4_ computed for all test images measured by D_1_-D_4_. The boxplots represent the results of this study using the Neural Network (NN) approach. Mean values are marked with ‘+’ and are further listed in the table including the standard deviation. For comparison purposes the supplementary error bars show the variability of manual landmark segmentations performed by ten experts. The comparative data are taken from a study conducted by Lohscheller et al. [6]. Again, means and standard deviations are listed in the table. All values are given in pixels.

The precisions *D*_*i*_ of the landmark positions *P*_*i*_ computed from the Neural Net segmentations for all 1,500 test images are given as boxplots in Fig 9. The corresponding mean values including the standard deviations are listed in the embedded table in the left column. For the two medial landmarks *P*_3,4_, located at the vocal edges, very high mean segmentation precisions of ${\bar{D}}_{3}\;=\;\left(0.59\;\pm \;0.66\right)\;\text{px}$ and ${\bar{D}}_{4}\;=\;\left(0.92\;\pm \;1.00\right)\;\text{px}$ were achieve. For the posterior and anterior landmarks mean precisions of ${\bar{D}}_{1}\;=\;\left(1.54\;\pm \;1.44\right)\;\text{px}$ and ${\bar{D}}_{2}\;=\;\left(5.76\;\pm \;9.70\right)\;\text{px}$ were obtained. Except for the landmark *P*_2_ the deviations between the segmentation results and the ground truth are in the range of one pixel.

## Discussion

Quantification of the VFs vibrational behavior is essential for the investigation and diagnosis of voice disorders. Up to the present the objective analysis of vocal fold dynamics bases, almost without exception, on the segmentation of the glottal area from laryngeal high-speed videos [12, 34, 35]. Techniques that are presently used thereto share the drawbacks of being computationally expensive or need frequently user-interaction [24, 25] which limits their application in e.g. large-scale studies. In this work we present for the first time a fully automatic segmentation not only of the glottal area but also of the vocal fold tissue itself. For the identification of the most suitable network architecture the performances of eighteen different Convolutional Neural Network configurations were investigated comparatively.

### Identification of the best performing network architecture

A refined version of the U-Net proposed by Ronneberger et al. [68] was used as basis architecture for high-speed videos segmentation. The following ordered selection strategy was used to identify the best performing network configuration. Initially, the best performing U-Net configuration concerning the number of levels, color space and image normalization was identified. Finally, the model was equipped with additional LSTM cells to investigate the benefit of integrating temporal information. In the following the respective results of the stepwise model selection will be discussed in detail.

(a) Statistical analysis revealed that the segmentation performance, measured by the Dice Coefficient (DC) of the glottis, significantly changes in respect to the number of U-Net levels. Up to five U-Net levels the segmentation performance increases steadily. Further increase beyond that, however, leads to a drop of the segmentation performance, cf. Fig 4(a). A reason for this might be the exceptional high number of additional feature maps generated for the higher levels, since the number of feature maps is exponentially related to the number of levels within the U-Net architecture. Thus, for a seven level network 64 feature maps of the first level (high spatial resolution) face 4096 features maps of the final level (low spatial resolution), the latter representing, however, just macroscopic image properties. Therefore, the ratio of the number of feature maps representing high and low spatial image characteristics, becomes increasingly unfavorable regarding fine image details, i.e. small glottal areas and thus worsens the segmentation performance. For high-speed video frames with a resolution of 256 × 256 pixels a five level U-Net turned out to be the best performing model.

(b) The segmentation performance of the U-Net is further sensitive to the applied color space. Statistical analysis showed significant changes of the Dice Coefficient between RGB, HSV and grayscale representations of the input images. Here, the U-Net configuration trained on RGB images showed the best performance, cf. Fig 4(b). This can be explained by considering the color of the anatomical structures. The intensity of the image is dominated by the red channel, showing different grades of saturation and brightness. As structure of interest the glottis appears much darker while the vocal fold (VF) tissue is lighter-colored exhibiting also a slightly different hues. Subjective evaluation of the separate channel intensity images revealed that this high contrast between glottis and surrounding structures is especially distinct in R- and G-channel and therefore might have enhanced NN performance over the NN trained on B-channel images. The NN trained on G-channel images showed a slightly higher mean and smaller variation of the Dice Coefficient (DC) compared to R-channel. Presumably this effect results from the Bayer pattern of the camera sensor, where the green channel is mapped by a higher spatial resolution. However, no improvement of segmentation quality based on a single RGB channel over full RGB images was found. In comparison to a pure intensity based grayscale representation, the coloring provides further valuable additional information which supports the proper distinction between glottis and vocal fold tissue. For the HSV color space, hue is encoded within an individual channel. Here, red as dominant color of soft tissue is represented by hue values around 0 and 2*π* respectively. Thus, slight variations of the coloring of red tissue lead to discontinuous measurements concerning the corresponding hue values which adversely affects the training process.

Our findings are in accordance with other studies which have shown that the used color space has an influence on the segmentation accuracy of a Convolutional Neural Network (CNN) [89, 90]. Cheng et al. provide an comprehensive overview on the use of different color spaces for various segmentation approaches, discussing also the drawbacks and advantages [91]. They conclude that there is no color space fitting best for all segmentation tasks and a convenient color space has therefore to be chosen for each segmentation task individually.

(c) Subsequent investigation of the potential impact of a z-normalization as data preprocessing step was done based on the ${\text{U-Net}}_{5}^{RGB}$. Other studies demonstrated the enhancement of NN performances by applying batch-normalization on various applications [61, 83, 92, 93]. They discuss that batch-normalization demands a sufficiently large batch-size such that the batch-statistics can be estimated adequately. In our experiments the implemented models were designed is such a way that even the U-Net with a maximum number of seven levels could be further equipped with additional LSTM layers at various positions. Due to the considerable size of the resulting models and computational limitations, our experiments were restricted to a batch-size of 10 images. In our experiments a positive affect of batch-normalization could not be found (cf. Fig 4c) which might be a result of the limited batch-size. To enhance the performance of the here presented NN based segmentation, further investigations might be done using other normalization-techniques, i.e. Instance Normalization [94], Layer Normalization [95], or Group Normalization [92].

(d) Finally, it was investigated if the segmentation of high-speed videos can be further improved by integrating B-CLSTM cells at different position into the NN to incorporate temporal information during the segmentation process. Statistical analysis revealed, that two of the four U-LSTM models indeed showed a significantly improved segmentation accuracy, cf. Fig 4(d). The best segmentation accuracy was obtained by the ${\text{U-LSTM}}_{5}^{CE}$ network. Here, not only the mean performance but likewise the segmentation stability could be improved which is reflected by the considerably reduced interquartile range of the Dice Coefficient (see boxplot). Intermittently appearing artifacts far away from the glottis were suppressed, leading to the presumption that the NN has learned mandatory spatial proximity of vocal folds and glottis. In this study segmentation performance could neither be improved by the use of GRU cells nor by data augmentation.

**Fig 10:**
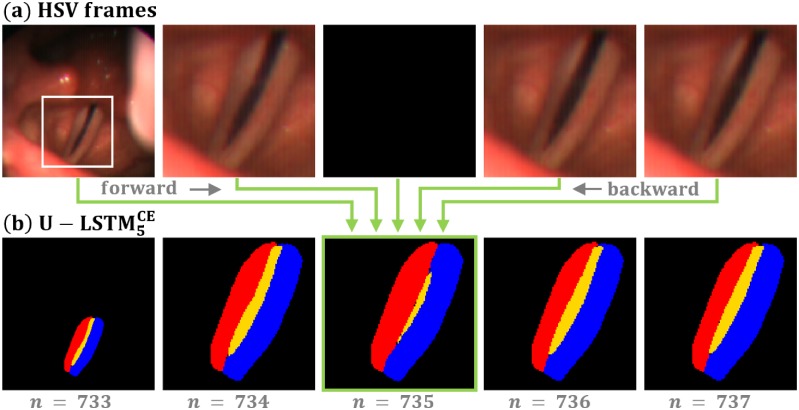
Exploiting temporal information by the introduced U-LSTM network to produce spatially and temporally consistent segmentation results. (a) Test dataset with an artificially included empty (black) image. (b) Segmentation result of the best performing ${\text{U-LSTM}}_{5}^{CE}$ model.

A further subjective rating of the segmentation results showed that the segmentation results became temporally more consistent. The LSTM based networks learned that besides a spatial there is also a temporal continuity of the segmented structures within a video sequence. Due to the use of bi-directional CLSTM cells the two segmented images located in the middle of the sequence receive information concerning the spatial localization of the glottis and of the vocal fold tissue from both temporal directions, which supports the segmentation process. Fig 10 demonstrates the benefit of incorporating temporal information using B-CLSTM cells. Within a video of the test dataset an (black) empty image is artificially introduced. The outcome of the segmentation using the ${\text{U-LSTM}}_{5}^{CE}$ network is shown in the lower row. Even for the empty image the Neural Network produced a temporally consistent segmentation outcome which is derived from the information propagated forward and backward from the remaining images of the sequence. The segmentation of the center image can be regarded as a bi-directional temporal interpolation which generates consistent results.

The architecture with LSTM cells placed just in the contracting path showed inferior segmentation. A reason for this might be that the propagated temporal information got lost due to max-pooling during downsampling in the contracting path of the $\text{U}-{\text{LSTM}}_{5}^{C}$. Likewise the add-on $\text{U}-{\text{LSTM}}_{5}^{AO}$ results do not reach the performance of the U-Net_5_. This might be because just the feature maps of the first level were applied to the LSTM-network and the information concerning the higher levels were not further considered.

### Accuracy of glottal area segmentation over time

Segmentation results were quantitatively evaluated according spatial and temporal segmentation congruency. High-quality segmentations were achieved throughout the entire sequences which is represented by a high conformity measured by the Dice Coefficient between the temporal structure of the segmented glottal area respectively the VFs and the Ground Truth (GT) segmentations, where the Convolutional Neural Network (CNN) tended to slightly overestimate the glottal area. Likewise, a study of Zijdenbos et al. found systematical differences between manual and automatic segmentation and explained their observation by the different perception of edges in medical images between humans and computerized segmentation approaches [96]. High DCs over the course of time indicate that no serious erroneous segmentations occurred. However, slightly lower Dice Coefficient (DC) values were observed around the closed state. These variations in the Dice Coefficient (DC) between different images within the sequence result not only from qualitatively differing segmentations, but are rather systematically influenced by the size of the segmented glottis. Smaller glottal areas lead systematically to lower Dice Coefficient (DC) values, which can be confirmed by a weak but significant correlation (*r* = 0.30, *n* = 1, 500, *p* = 0) between the size of the glottal area and the Dice Coefficient (DC). Therefore, a single misclassified pixel in a frame around the closed state has a higher impact on the resulting Dice Coefficient (DC) than around opened state. Another reason for the variations of the Dice Coefficient (DC) is, that low contrast as well as blurring around the closed state makes a differentiation between *glottis* and surrounding *vocal fold* pixels more difficult. Therefore, recordings from subjects with a pronounced glottal closure, which is characteristic particularly for healthy voices, show a higher variability of the Dice Coefficient (DC) compared to vibrations exhibiting insufficient glottal closure. The influence of glottal closure on the DC variability is exemplarily shown in Fig 6. Additionally, the NN tends to segment the vocal fold (VF) edges slightly smoother compared to the Ground Truth (GT) segmentation, i.e. for the in Fig 6(b) shown carcinoma. This can be explained by the fact that the NN has learned rather a smooth shape of the vocal fold (VF) edges caused by the lower scale levels.

With a median of $\tilde{DC}\;=\;0.88$ correctly classified glottis pixels for *all* investigated images, the trained Convolutional Neural Network (CNN) has been proven to be suitable for an automated glottis segmentation as indicated by values of *DC* > 0.7, which are generally seen as an *“excellent agreement”* [96, 97] and surpasses furthermore the segmentation accuracy of $\tilde{DC}\;=\;0.767$ achieved by Schenk et al. using salient regions and 3D geodesic active contours [47]. Regarding the segmented glottis, between the three investigated groups variation of the mean DCs was smaller than Dice Coefficient (DC) variation within the groups, indicating that pathologies do not affect the overall segmentation performance. Segmentation accuracy for both VFs was even higher, resulting on the one hand from the higher number of pixels for this classes and on the other hand from their temporal stability, since the VFs do not change their outer shape rapidly during short time intervals.

### Precision of anatomical landmark positions

To further evaluate the accuracy of the obtained segmentations, the precisions at four anatomically relevant landmark positions *P*_1_-*P*_4_ were investigated in detail. The landmarks were chosen in accordance with a comparable study conducted by Lohscheller et al. which is up the present the only study dealing with a comprehensive evaluation of the accuracy of high-speed video segmentations [6]. In that study the landmarks were manually segmented by a group of ten experts to obtain ground truth segmentations which can be regarded as gold standard. There it was shown that all manual segmentations underlie a comparable variability of about 3 px which can be regarded as a general imprecision of the gold standard.

In our study, for all landmark positions, except for *P*_2_, the mean deviations *D*_*i*_ obtained from the NN segmentations are far below the mean deviations of the expert group. Particularly, for the landmarks *P*_3_ and *P*_4_ a very high precision of less than one pixel was obtained which highly outperforms the gold standard accuracy. For the anterior landmark position *P*_2_ a mean precision of ${\bar{D}}_{2}^{NN}=5.76\;\text{px}$ was achieved which is a bit more imprecise compared to the expert group. This is caused if small ventral parts of the glottal area, which partially arise during the closing or opening phase of a vibration cycle, are not properly segmented by the Neural Network as exemplarily shown in Fig 8. This results into high deviation outliers of up to 63 px that strongly effect the mean value. Considering the median, which is more robust against outliers, a value of ${\tilde{D}}_{2}^{NN}\;=\;2.50\;\text{px}$ was obtained which is comparable to the manual segmentation accuracy of the expert group.

Due to the lack of further quantitative studies a concrete comparison to other approaches could not be performed. Since a public available reference dataset of annotated laryngeal high-speed videos would be useful for a profound quantitative comparison of different approaches in future the here used dataset including the Ground Truth (GT) segmentations will be provided freely for all scientific groups. Further information can be found at www.hochschule-trier.de/go/quantitative-laryngoscopy.

Besides the segmentation of the glottis we further achieved for the first time an automatic and precise segmentation of the vibrating vocal fold tissue from laryngeal high-speed videos. Knowledge about the length and width of the vocal folds might be helpful in a clinical setting to derive indirect information about the internal state of vocal fold tension, which is relevant to assess particularly muscle tension dysphonia. Furthermore, subsequent analysis of the color and texture of vocal fold tissue can deliver valuable information about inflammatory processes or organic changes.

Based on the results of the comparable study it can be stated that the here proposed procedure allows a very stable and high quality segmentation of both the glottal area and the vocal folds. Once the Neural Network is trained, high-speed video (HSV) recordings can be segmented without any user-interaction, which is an essential prerequisite for the use in clinical practice. Parallelization of the segmentation process likewise enables the analysis of even long high-speed video (HSV) sequences comprising several thousand frames. Although data augmentation showed no benefit on segmentation accuracy, further optimization and generalization of the obtained model might be achieved by enlarging the dataset comprising a higher number of subjects, ideally acquired at several clinics with different high-speed camera systems. Further, an improvement of the network architecture itself might facilitate segmentation performance of the Neural Network as well as potentially speed-up training and testing. Hence, future work will focus on the investigation of alternative models, i.e. the One-Shot Video Object Segmentation (OSVOS) Convolutional Neural Network (CNN) [98], Dilated Residual Networks [99] models or further variations of the U-Net like proposed by Li et al. [100], or the Recurrent Residual U-Net (R2U-Net) [101]. Another approach to achieve better generalization might be the construction of a model from a heterogeneous collection of Neural Networks (NNs) as suggested by Kaminitas et al. [102], since the so trained NN is insensitive to independent failures of the individual Convolutional Neural Network (CNN) components.

## Conclusion

The precise segmentation of the VFs edges from high-speed video (HSV) is an essential prerequisite for a quantitative analysis of VFs vibrations. In this work, for the first time a fully automated segmentation of the glottis and the vibrating vocal fold tissue was presented using a deep Convolutional LSTM Network. It could be shown that integrating temporal information into the segmentation process, by adding bi-directional convolutional LSTM cells into a refined U-Net architecture, significantly improves the segmentation performance. The results of an extensive evaluation of the segmentation accuracy reveal that, both in pathologic as well as in healthy subjects, the obtained segmentation precisions are comparable to manual segmentations or even superior. The comparison with other approaches is currently hardly possible due to the lack of appropriate reference datasets. To resolve this deficiency the data used in this study will be made freely available on demand. Since the procedure is fast, fully automated and does not need any user-interaction, the presented approach holds the potential to facilitate a quantitative analysis of vocal fold dynamics in clinical practice.

## Supporting information

S1 Animation Laryngeal high-speed video sequence of a healthy subjects vocal fold vibration. (a) Glottal area segmentation congruency over time. Corresponding Ground Truth and Neural Network segmentation for glottis and vocal folds are shown. The size of the segmented glottal area as well as the achieved segmentation congruency over time measured by the Dice coefficient is illustrated underneath (black: glottis, red: right VF, blue: left VF). (b) Precision of anatomical landmark positions. Landmarks indicated by ‘+’. Corresponding deviations over the course of time are displayed underneath (yellow: P_1_, green: P_2_, red: P_3_, blue: P_4_).
(GIF)

S2 Animation Laryngeal high-speed video sequence of a pathologic subjects (carcinoma) vocal fold vibration. (a) Glottal area segmentation congruency over time. Corresponding Ground Truth and Neural Network segmentation for glottis and vocal folds are shown. The size of the segmented glottal area as well as the achieved segmentation congruency over time measured by the Dice coefficient is illustrated underneath (black: glottis, red: right VF, blue: left VF). (b) Precision of anatomical landmark positions. Landmarks indicated by ‘+’. Corresponding deviations over the course of time are displayed underneath (yellow: P_1_, green: P_2_, red: P_3_, blue: P_4_).
(GIF)
