# Supplementary figures and images for: Fully automatic segmentation of glottis and vocal folds in endoscopic laryngeal high-speed videos using a deep Convolutional LSTM Network
Source: PLoS One. 2020 Feb 10;15(2):e0227791. doi: 10.1371/journal.pone.0227791 (PMC7010264; doi:10.1371/journal.pone.0227791)

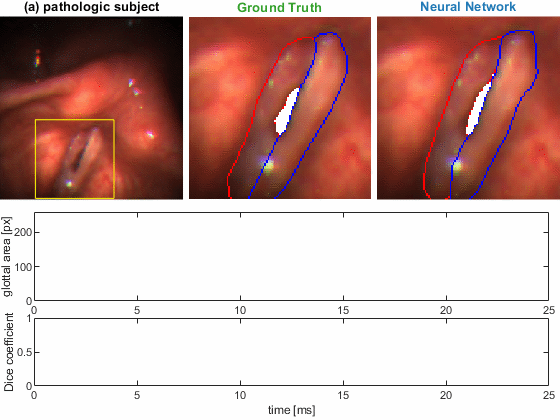

Supplement: S1 Animation — (a) Glottal area segmentation congruency over time. Corresponding Ground Truth and Neural Network segmentation for glottis and vocal folds are shown. The size of the segmented glottal area as well as the achieved segmentation congruency over time measured by the Dice coefficient is illustrated underneath (black: glottis, red: right VF, blue: left VF). (b) Precision of anatomical landmark positions. Landmarks indicated by ‘+’. Corresponding deviations over the course of time are displayed underneath (yellow: P1, green: P2, red: P3, blue: P4). (GIF) [file pone.0227791.s001.gif]

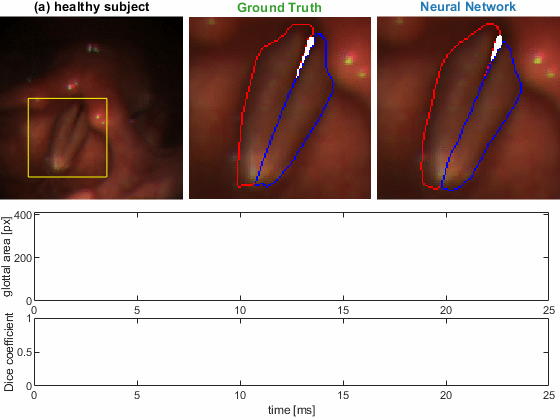

Supplement: S2 Animation — (a) Glottal area segmentation congruency over time. Corresponding Ground Truth and Neural Network segmentation for glottis and vocal folds are shown. The size of the segmented glottal area as well as the achieved segmentation congruency over time measured by the Dice coefficient is illustrated underneath (black: glottis, red: right VF, blue: left VF). (b) Precision of anatomical landmark positions. Landmarks indicated by ‘+’. Corresponding deviations over the course of time are displayed underneath (yellow: P1, green: P2, red: P3, blue: P4). (GIF) [file pone.0227791.s002.gif]
